# The Biophysical Probes 2-fluorohistidine and 4-fluorohistidine: Spectroscopic Signatures and Molecular Properties

**DOI:** 10.1038/srep42651

**Published:** 2017-02-15

**Authors:** Chandana Kasireddy, Jonathan M. Ellis, James G. Bann, Katie R. Mitchell-Koch

**Affiliations:** 1 Department of Chemistry, Wichita State University, 1845 Fairmount Street, Wichita, KS 67260-0051, CV4 7AL, United States

## Abstract

Fluorinated amino acids serve as valuable biological probes, by reporting on local protein structure and dynamics through ^19^F NMR chemical shifts. 2-fluorohistidine and 4-fluorohistidine, studied here with DFT methods, have even more capabilities for biophysical studies, as their altered *pK*_*a*_ values, relative to histidine, allow for studies of the role of proton transfer and tautomeric state in enzymatic mechanisms. Considering the two tautomeric forms of histidine, it was found that 2-fluorohistidine primarily forms the common (for histidine) τ-tautomer at neutral pH, while 4-fluorohistidine exclusively forms the less common π-tautomer. This suggests the two isomers of fluorohistidine can also serve as probes of tautomeric form within biomolecules, both by monitoring NMR chemical shifts and by potential perturbation of the tautomeric equilibrium within biomolecules. Fluorine also enables assignment of tautomeric states in crystal structures. The differences in experimental *pK*_*a*_ values between the isomers was found to arise from solvation effects, providing insight into the polarization and molecular properties of each isomer. Results also encompass ^13^C and ^19^F NMR chemical shifts, from both tautomers of 2-fluorohistidine and 4-fluorohistidine in a number of different environments. This work can serve as a guide for interpretation of spectroscopic results in biophysical studies employing 2-fluorohistidine and 4-fluorohistidine.

Histidine is one of the most chemically versatile amino acids, with the neutral imidazole ring having the potential to act as Lewis acid, Lewis base, hydrogen bond donor, and hydrogen bond acceptor. Further adding to its chemical diversity is the fact that the imidazole side chain of histidine can exist in two tautomeric states: the τ (N3-H or N_ε2_-H) and π (N1-H or N_δ1_-H) tautomers, as shown in Fig. 1. Likely as a consequence of this chemical “flexibility”, histidine is present in ~50% of enzyme active sites^1^. The *pK*_*a*_ (~6) of the imidazole ring in histidine is close to physiological pH, which makes it an important amino acid in protein function^2^,^3^,^4^,^5^. The fluorinated analogues of histidine, which exist as two different regioisomers, have dramatically lower *pK*_*a*_ values than canonical histidine: ~1 (aqueous) for 2-fluorohistidine (2-FHis) and ~2 (aqueous) to >3 (proteins) for 4-fluorohistidine (4-FHis). This paper focuses on the properties of 2-FHis and 4-FHis, shown in both tautomeric forms in Fig. 1, including the origin of the differences in *pK*_*a*_ between the two regioisomers. It also serves as a guide for use and spectroscopic interpretation of fluorohistidine in biophysical studies.

Multiple methods for the synthesis of fluorohistidines have been developed^6^,^7^,^8^,^9^,^10^. The ability to incorporate these analogues of histidine into proteins has facilitated mechanistic studies of proton transfer (discussed below) and characterization of protein structure using ^19^F-NMR^11^,^12^,^13^,^14^,^15^,^16^,^17^,^18^,^19^,^20^. Interestingly, the fluorine chemical shifts of 2- and 4-fluorohistidine exhibit anomalous pH-dependencies: 2-FHis is deshielded upon protonation, while the ^19^F in 4-FHis becomes more shielded at low pH^21^. We recently used electronic structure calculations to show that electron delocalization/localization appears to give rise to the differences in ^19^F shielding^22^.

Both 2-FHis and 4-FHis have been successfully incorporated into proteins via chemical or biosynthetic methods^3^,^23^,^24^,^25^,^26^,^27^,^28^. Fluorohistidine-labeling of proteins has provided valuable structural and mechanistic information in several instances^23^,^25^,^29^,^30^, and has great potential for future work. Studies by Eichler *et al*. showed that 2-FHis, and to a lesser extent 4-FHis, could be biosynthetically incorporated through protein expression in *E. coli*^30^. Prior to this, Jackson *et al*., in an amazing effort, chemically synthesized ribonuclease A, incorporating 4-fluorohistidine to show that the pH-dependence of enzyme activity could be shifted to lower pH values^29^. In crystallography, the fluorine electron density at the 2 or 4 position of the imidazole ring in histidine provides a marker so that carbon and nitrogen nuclei can be distinguished. Based on this information and inferred hydrogen bonding sites from other heavy atoms, tautomeric forms and nitrogen identities (N1 vs. N3) of histidine can be assigned with low ambiguity in protein crystal structures, as seen in work by Bann and co-workers for anthrax protective antigen labeled with 2-fluorohistidine^25^.

The potential for different biophysical studies based on the molecular and spectroscopic properties of 2-fluorohistidine and 4-fluorohistidine is discussed in this paper. In particular, the fluorohistidine isomers are poised to be powerful probes of histidine protonation state (tautomeric form) within proteins. Our calculations indicate that the τ-tautomer of 2-FHis is favored in the neutral state, while the π- tautomer is strongly favored for 4-FHis. Meanwhile, in canonical histidine, the τ-tautomer is more stable than the π-tautomer in the ratio of ~4:1 at pH = 8^31^. In metalloproteins, different tautomeric forms of histidine interact with metals, such as zinc^32^,^33^, and local environments can shift the tautomeric equilibrium of histidine side chains through hydrogen bonding. Tautomerism appears to play a role in some enzymatic mechanisms^34^,^35^,^36^,^37^,^38^,^39^,^40^. For instance, the catalytic activity of human carbonic anhydrase II (hCAII) was investigated by Shimahara *et al*., revealing that the rate of proton transfer in the catalytic mechanism is highly efficient through a histidine tautomerization pathway at neutral pH^34^.

Traditionally, studies of histidine in protein function have used NMR spectroscopy to assign protonation state^21^,^31^,^41^,^42^,^43^,^44^,^45^,^46^,^47^,^48^,^49^,^50^. The tautomeric states of histidine can be distinguished by ^15^N NMR isotropic and anisotropic studies^51^ and C4 and C5 (C_δ2_ and C_ϒ_) chemical shifts^43^. NMR experiments indicate rapid equilibration between tautomers^52^, and recent NMR studies of proteins have revealed hidden tautomeric states of histidine in transient protein conformations^43^. Work by Scheraga^41^, Oldfield and co-workers^46^ showed the utility of calculated and experimental ^13^C NMR shifts to evaluate tautomeric forms, and indicated that tautomeric form and chemical shifts of histidine are highly dependent on intramolecular hydrogen bonding. Results from DFT calculations presented below enable spectroscopic identification (via ^19^F, ^13^C chemical shifts) of the τ- and π-tautomers of 2- and 4-FHis in different environments (*i.e.,* with or without intramolecular hydrogen bonding, in different dielectric environments). The nomenclature used to describe atoms in the imidazole ring throughout this paper is illustrated in Fig. 2a, alongside Fig. 2b, which shows labels used to identify the atoms of the histidine side chain in other literature reports.

## Results

The tautomeric stabilities of 2-fluorohistidine and 4-fluorohistidine incorporated into the Gly-FHis-Gly tripeptide were calculated using multiple methods. These energy differences were found to be similar across a survey of electronic structure methods, as shown in Table S1 of Supplementary Information. Results discussed below are from BHandHLYP density functional, 6–311++G(d, p) basis set, and CPCM implicit solvation. The BHandHLYP functional has been recommended by Yu *et al*. for amino acids after a systematic study of multiple DFT methods^53^, and it can be seen here that it provides theoretical results for the fluorohistidine isomers that are in very good agreement with experimental data.

For 2-fluorohistidine, the τ-tautomer is more stable than the π-tautomer by 1.70 kcal/mol, resulting in a Boltzmann ratio of 94:6 τ:π tautomer at 310 K. The energy differences between the tautomers of 4-fluorohistidine are larger, with the π-tautomer being more stable than the τ-tautomer by an energy difference of 4.85 kcal/mol. The crystal structure of 2-fluorohistidine indicates protonation at the τ-tautomer position^54^. Although XRD data cannot conclusively give proton locations, the N3-C2 bond length compared to the N1-C2 bond length indicates protonation at N3 (τ-tautomer). In the case of 4-fluorohistidine, the stability of the π-tautomer can be rationalized by the position of electron-withdrawing fluorine in the imidazole ring: the nitrogen nearest to F4 is less basic (N3, unprotonated in the π tautomer), while the N1 position has higher electron density and is more basic.

**Figure 3:**
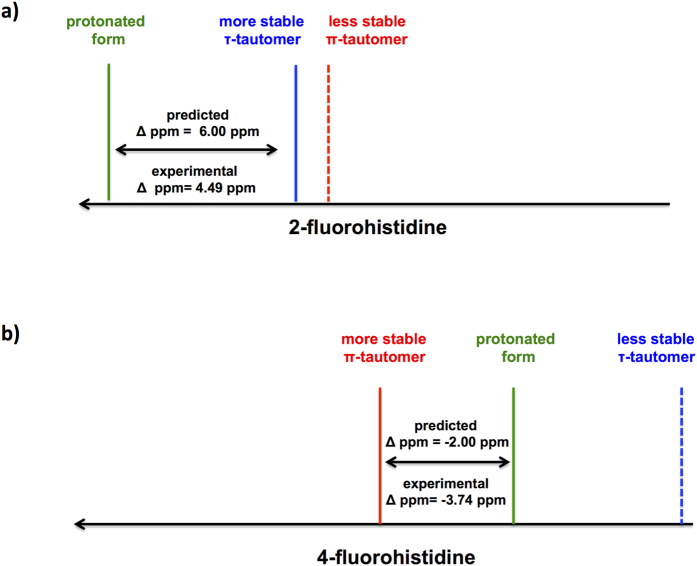
Calculated fluorine chemical shifts for the τ and π-tautomers and protonated forms of zwitterionic (**a**) 2-fluorohistidine and (**b**) 4-fluorohistidine.

Figure 3 illustrates how the experimental and calculated ^19^F NMR spectra support the calculated tautomeric stabilities, by plotting experimental titration data for 2-FHis and 4-FHis^21^ alongside the calculated ^19^F chemical shifts of both tautomers and protonated forms of the fluorohistidine isomers. In experiments, 2-fluorohistidine showed a downfield shift of 4.49 ppm upon protonation (from 59.80 ppm at pH ~7 to 64.29 ppm at pH ~1). In the same way, calculations for zwitterionic 2-fluorohistidine show deshielding of 6.0 ppm upon protonation for the τ-tautomer, while the signal shifts downfield by 9.7 ppm when the π-tautomer is protonated. For 4-fluorohistidine, the calculated chemical shift upon protonation of the π-tautomer is 2 ppm upfield (*cf.* 3.74 ppm upfield experimentally). In contrast, the calculated chemical shift change of the τ-tautomer (calculated to be highly unstable) is 8.6 ppm *downfield*, contrary to the direction of the ^19^F chemical shift observed in the experimental titration.

In summary, the simulated ^19^F NMR chemical shifts upon acid titration are in good agreement with experimental results for the tautomers that are calculated to be more stable. However, the less stable tautomers show divergence from experimental results in the simulated acid titration, with 4-FHis giving shifts in the opposite direction and 2-FHis showing a bigger change in chemical shifts than what is observed. Experimental support for the strong preference of 4-fluorohistidine for the π-tautomer is provided in a temperature-dependent ^19^F NMR spectrum, acquired in water (Fig. S2 of Supplemental Information). The thermal broadening and shifting of the ^19^F chemical shift as 4-fluorohistidine is heated to 75 °C is similar to the changes in chemical shift observed in the reference compound (trifluoroacetic acid or TFA). When the chemical shift of 4-fluorohistidine is calculated relative to TFA at every temperature point, the peak shifts by less than 0.1 ppm. If the relative energies of the 4-fluorohistidine tautomers were closer together (on the order of 1 kcal/mol or less), chemical exchange would lead to an upfield shift of the ^19^F peak as temperature increases, but there is no indication of this in the spectrum.

### What are the effects of environment on tautomeric stabilities and fluorine chemical shifts?

Calculations were carried out on the Gly-FHis-Gly tripeptides in different dielectric environments, using CPCM-modeled acetonitrile and tetrahydrofuran (THF), to see the effect on tautomeric stabilities and fluorine chemical shifts. The results are provided in Table 1. Moving from an aqueous environment to a less polar environment slightly shifted the tautomeric equilibrium of 2-fluorohistidine toward the π-tautomer, with a relative stability of ~1.35 kcal/mol higher than the τ-tautomer in acetonitrile and THF (compared to 1.70 kcal/mol in water). These relatively low energy differences, in combination with a hydrogen-bonding environment that favors stabilization of the π-tautomer over the τ-tautomer, are supported by experimental results. In the crystal structure of 2-fluorohistidine labeled anthrax protective antigen, the π-tautomer of 2-fluorohistidine is indicated to be more stable in some interior sites (*i.e.* 2-FHis616 in the hydrophobic core of domain 4, stabilized by a hydrogen bond to N3)^27^. This is similar to histidine, which favors the π-tautomer in some protein environments. In less polar environments, the ^19^F chemical shifts of both tautomers of 2-fluorohistidine becomes slightly more shielded, with changes on the order of 0.6 ppm.

For 4-fluorohistidine, the relatively hydrophobic environment of THF shifts the tautomeric equilibrium slightly towards the τ-tautomer (3.91 kcal/mol higher than the π); acetonitrile shows a small change (4.75 kcal/mol, relative to 4.85 kcal/mol in water). Dielectric is seen to have very little influence on the ^19^F chemical shift of the τ-tautomer (less than 0.1 ppm), while less polar environments deshield the ^19^F nucleus of the 4-FHis π-tautomer from 19.21 ppm in water to 19.31 in acetonitrile and 20.16 ppm in THF. Such a “reverse” or abnormal fluorine chemical shift is discussed more in depth in our recent publication^22^.

#### Effects of intramolecular hydrogen bonding on fluorine chemical shifts and tautomeric stabilities

“Capped” histidine (*N-*acetyl-FHis-O-methyl ester or NAc-FHis-OMe), which has no intramolecular hydrogen bonds with the imidazole ring, was compared to zwitterionic histidine, for which the lowest-energy conformation in implicitly-modeled solvent has hydrogen bonds between the imidazole ring (side chain) and amino or carboxylate moieties of the amino acid. Figure S1 of Supplementary Information shows the lowest-energy optimized geometries of all tautomers of the capped and zwitterionic fluorohistidines. The relative energies and ^19^F chemical shifts for these species are presented in Table 2.

For 2-fluorohistidine, the effects of intramolecular hydrogen bonding are minor: the fluorine chemical shifts are affected more in the τ-tautomer (deshielded ~0.4 ppm with hydrogen bond) than the π-tautomer (increased shielding of ~0.1 ppm with hydrogen bond). Meanwhile, the relative tautomeric stabilities of 2-fluorohistidine change by <0.2 kcal/mol with the intramolecular hydrogen bond. In contrast, 4-fluorohistidine appears to be more sensitive to hydrogen bond effects: the fluorine chemical shift of the stable π-tautomer has a difference of >3 ppm between forms, with hydrogen-bonded zwitterionic 4-fluorohistidine being more shielded at 16.89 ppm than the non-hydrogen-bonded capped 4-fluorohistidine at 20.21 ppm. This is another “reverse” effect in the fluorine chemical shift of 4-FHis, since hydrogen bonding would be expected to inductively shift electron density away from the fluorine. Considering tautomeric stability, hydrogen bonding contributes 0.9 kcal/mol stabilization to the τ-tautomer of 4-fluorohistidine; yet the π-tautomer remains substantially more stable, by ~4 kcal/mol.

Finally, substitution at C5 of the fluoroimidazole was used to determine the sensitivity of tautomeric stabilities and fluorine chemical shifts to structure. C5 substitution can probe the sensitivity of fluorine chemical shifts to changes in primary and secondary structure, both of which give rise to changes in the wave function near the nucleus of interest. This is described by Oldfield and co-workers as a short-range contribution to chemical shifts, which propagates through the bonding framework^55^. In this case, the tripeptide was compared with 2F- and 4F-(5-methyl)-imidazole and -(5-trifluoromethyl)-imidazole, providing information about electron-donating and –withdrawing effects, respectively. The relative tautomeric stabilities and fluorine chemical shifts for this series are presented in Table 3.

As can be seen in Table 3, the relative stabilities of tautomers are dramatically affected by substitution at C5. In the case of 2-fluoro-(5-methyl)-imidazole, electron-donating substitution substantially stabilizes the π-tautomer, resulting in isoenergetic tautomers, while the electron-withdrawing CF_3_ group strongly destabilizes the π-tautomer, relative to the τ-tautomer. The effect is opposite for 4-fluorohistidine: electron-donating substituents further stabilize the π-tautomer, while electron-withdrawing leads to stabilization of the τ-tautomer. However, the energy differences between 4-fluoroimidazole tautomers always remain such that the τ-tautomer remains <1% population in equilibrium at biological or room temperatures.

The fluorine chemical shifts of 4-fluorohistidine are shown to depend dramatically on C5 substitution: electron donation causes an upfield shift (~5 ppm for both tautomers), while electron-withdrawing shifts the signal downfield (>15 ppm). The ^19^F NMR spectra are not as dramatically influenced by C5 substitution for 2-fluorohistidine, however. The differences between tripeptide and (5-methyl)-imidazole are slight (<0.2 ppm), and CF_3_, a rather large perturbation on electronic structure, shifts the signal 3 ppm and 6.7 ppm for the τ- and π-tautomers, respectively.

#### The origin of *pK*_*a*_ differences between fluorohistidine isomers

The measured *pK*_*a*_ values for aqueous fluorohistidine isomers indicate that 2-fluorohistidine has a lower *pK*_*a*_ than 4-fluorohistidine (~2 for 4-FHis and ~1 for 2-FHis)^21^. The differences in relative tautomeric stabilities between 2-fluorohistidine and 4-fluorohistidine give rise to an entropic factor in the differences in *pK*_*a*_. Neutral 2-FHis is entropically stabilized by its thermally-accessible (tautomeric) states, whereas protonated imidazoliums and netural 4-fluorohistidine are not. Using the Boltzmann definition of entropy, the molar free energy of neutral fluorohistidine is reduced by RT ln 2 (drop in *pK*_*a*_ of 0.3) when the two tautomers are isoenergetic (degeneracy = 2). In other cases, the closer the tautomers are in energy (and, hence, the closer their “microscopic” or individual *pK*_*a*_ values)^47^,^56^, the greater the reduction in observed *pK*_*a*_ value relative to microscopic *pK*_*a*_. In summary, the *pK*_*a*_ of 2-fluorohistidine is predicted to be reduced by tautomerism, whereas the same effect is not predicted in 4-fluorohistidine.

Next, structural contributions to *pK*_*a*_ differences between the most stable tautomers of 2-FHis and 4-FHis were considered. The origins are not immediately obvious by considering resonance and inductive effects. To study this computationally, the gas phase energies of capped 2-fluorohistidine and 4-fluorohistidine, and their conjugate acids were calculated and are presented in Table S2 of SI. The gas phase basicities (E_conjugate acid_ − E_neutral_) of the two isomers were found to be virtually identical: within 0.2 kcal/mol of one another. Therefore, solvation appears to be a driving force for the differences in *pK*_*a*_. Indeed, calculations confirm this, with protonation of aqueous 4-fluorohistidine being more favorable than protonation of 2-fluorohistidine by a difference of 1.4 kcal/mol. This is in very good agreement with the *pK*_*a*_ difference of ~1–2 reported for the isomers^21^, since a *pK*_*a*_ difference of 1 corresponds to a free energy difference of 1.37 kcal/mol at 300 K.

Solute polarity is a favorable driving force for solvation in water. Considering the apparent polarity of fluorohistidines, by examining their dipole moments (Table 4), it would appear there is a greater solvation driving force for protonation of 2-fluorohistidine than 4-fluorohistidine. In fact, if approximating solvation energy by the Onsager model, in which ΔG_solvation_ is proportional to μ^2^ within the same solvation environment^57^, one would predict the solvated protonation of 2-fluorohistidine to be 1.5 times more favorable. This would give rise to a higher *pK*_*a*_ for 2-fluorohistidine than 4-fluorohistidine, the opposite of what is observed and calculated. If the dipole moments cannot explain trends in solvation, the charge distributions must hold the answer.

**Figure 4:**
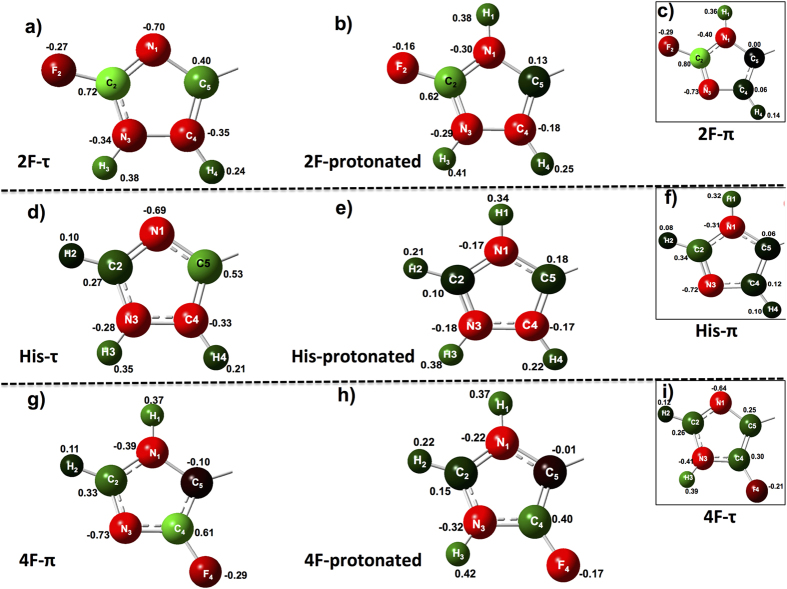
Calculated charges for imidazole side chains in Gly-FHis-Gly peptides for (**a**) τ-tautomer of 2-fluorohistidine (**b**) protonated form of 2-fluorohistidine (**c**) π-tautomer of 2-fluorohistidine; (**d**) τ-tautomer of histidine (**e**) protonated form of histidine (**f**) π-tautomer of histidine; (**g**) π-tautomer of 4-fluorohistidine (**h**) protonated form of 4-fluorohistidine (**i**) τ-tautomer of 4-fluorohistidine.

The key to understanding how solvation energies affect *pK*_*a*_ lies in the unexpectedly favorable solvation energy of neutral 2-fluorohistidine, which is very similar to ΔE_solv_ of 4-fluorohistidine (given in Table S2 of Supplementary Information), in spite of dissimilar dipole moments. Of course, the value of ΔE_solv_ depends on the charge distribution within the molecule. The charges for the imidazole side chains of protonated and neutral fluorohistidine tripeptides are presented in Fig. 4. The charges are colour-coded, with green negative charges, red positive charges, and dark shading for near-neutral atoms.

The salient difference between 2-fluorohistidine (τ-tautomer) and all other species is the polarization of the C4-H4 bond, which has a carbon charge of −0.35 and hydrogen charge of +0.24. This charge difference is somewhat less, but on the order of, the N-H bond polarity seen in both fluorohistidine imidazole N-H bonds. The other charges, in the lone pair nitrogens, C-F bonds, and N-H bonds, are fairly similar among all species. Thus, the favourable solvation of the additional polar bond (C4-H4) in the imidazole ring (τ-tautomer only) of neutral 2-fluorohistidine appears to reduce the driving force in ΔE_solv_ for protonation, making 2-fluorohistidine more acidic than 4-fluorohistidine.

It is interesting to note that calculations with the same level of theory on canonical histidine indicate that the properties of 2-fluorohistidine track very well with those of histidine. The C4-H4 bond of histidine is also polar (charges of −0.275 for C and +0.201 for H) in the τ-tautomer, while the same bond in the π-tautomer is nonpolar (like 2-fluorohistidine). Recently, Kubarych, Pecoraro, and co-workers reported on the polarity of the Cδ2-H bond (on a Zn-bound histidine residue) in a *de novo* peptide, pointing out that the QM results are substantiated by microwave spectroscopy of imidazole^58^,^59^. It is seen below that the higher charge density of the C4 (C_δ2_) carbon corresponds with a more shielded ^13^C NMR shift for this carbon in the τ-tautomer imidazole ring in both histidine and 2-fluorohistidine.

##### Spectroscopic signals of the tautomers of 2-fluorohistidine

The results presented above indicate the π-tautomer of 4-fluorohistidine is so much more stable than the τ-tautomer that the τ-tautomer is not predicted to be observed (<1%). However, calculations indicate small relative energy differences between the τ- and π-tautomers of 2-fluorohistidine, and both tautomers of 2-fluorohistidine have been observed in the crystal structure of labelled anthrax protective antigen^27^. Therefore, the discussion below focuses primarily on distinguishing among the two tautomers of 2-fluorohistidine in solution. ^13^C NMR chemical shifts for 2-fluorohistidine and 4-fluorohistidine in the Gly-FHis-Gly tripeptide are presented in Table 5.

Previous work by Oldfield^46^, Scheraga^41^, and co-workers have shown that the tautomeric forms of canonical histidine depend strongly on peptide environment (as seems to be the case for 2-fluorohistidine). Moreover, in their work, theoretical methods used to predict tautomeric fractions (relative stabilities) and the ^13^C NMR chemical shifts of each tautomer have been shown to be reliable. Oldfield and co-workers used a large data set of histidine-containing peptides and provided method-dependent scaling factors for each of the carbons in the histidine imidazole ring.

As is seen in canonical histidine, the C2 chemical shift of 2-fluorohistidine and 4-fluorohistidine are invariant with tautomeric form, while the C4 and C5 chemical shifts distinguish tautomers^41^,^46^. The C2 shift for both tautomers of 2-fluorohistidine is ~157 ppm: 157.4 ppm for the τ-tautomer and 156.9 ppm for the π-tautomer. The values of the C4 and C5 chemical shifts exhibit a wide spread for the τ-tautomer, with chemical shifts at 118.3 (C4/Cδ2) and 142.8 (C5/C_ϒ_) ppm. In contrast, the C4 and C5 chemical shifts are closer together in the π-tautomer, at 129.7 and 133.5 ppm, respectively. This range of chemical shifts between the 2-fluorohistidine tautomers (~11 ppm for C4 and ~10 ppm for C5) is on the order of the range observed for canonical histidine tautomers in a Gly-His-Gly peptide studied by Scheraga and co-workers^41^.

The range of chemical shifts for the carbons in the two tautomers of 2-fluorohistidine directly reflect the charge distribution on the carbons. The C4 carbon of the τ-tautomer is significantly more shielded (118.3 ppm) than the other carbons, corroborating its calculated negative charge (−0.4). As expected the C2 carbon bonded to fluorine is the most deshielded (~157 ppm for both tautomers), reflecting the polarity of the C-F bond. The polarization of C4 in the τ-tautomer coincides with polarization of the adjacent carbon, C5, which a relatively deshielded shift at 142.8 ppm (reflected in the calculated charge of +0.40). In contrast, there appears to be little polarization of C4 and C5 in the imidazole ring of the less polar π-tautomer, with chemical shifts that are close together, at 129.7 (C4) and 133.5 ppm (C5).

An experimental ^13^C NMR spectrum was acquired in deionized water for zwitterionic 2-fluorohistidine in water, and the imidazole ring shifts are as follows: 127.9 ppm, 113.6 ppm, 150.0 ppm for C5, C4 and C2 carbons respectively. The C5, C4, and C2 shifts for capped 2-fluorohistidine (close in structure to the aqueous zwitterion), provided in Table S3 of Supplementary Information, are calculated to be: 141.4 ppm, 118.9 ppm, 157.1 ppm respectively. Using a scaling factor approach in the same vein as Oldfield and co-workers, recommended scaling factors for BHandHLYP/6–311 ++ G(d, p) calculated ^13^C chemical shifts of fluorohistidines are as follows: 1.11, 1.05, 1.05 (C5, C4, C2). This is quite good agreement, indicating that the BHandHLYP functional may be a fairly reliable method for ^13^C chemical shifts (as it is for ^19^F).

Although ^13^C chemical shifts should not be needed to differentiate the tautomers of 4-fluorohistidine, given that the τ-tautomer is considerably more unstable, it is worth remarking on the predicted spectra. The π-tautomer of 4-fluorohistidine exhibits a broader spread in carbon chemical shifts than the τ-tautomer. We have recently published the experimental ^13^C spectrum of aqueous 4-fluorohistidine, and calculated values of the π-tautomer are in good agreement^60^. The C5 carbon has a relatively shielded nucleus at 101 ppm, and C4 (attached to fluorine) is the most deshielded at 152 ppm. The carbon chemical shifts again correlate with polarity and calculated charges: the C-F bond in the π-tautomer is more polar than the τ-tautomer, with a positive charge on the C4 carbon of +0.60 (−0.29 on F). The most shielded carbon (C5) is upfield at 101 ppm, corroborating a calculated charge of −0.10.

## Discussion

It has been observed that typically, biosynthetic incorporation of 2-fluorohistidine is more successful than biosynthetic incorporation of 4-fluorohistidine^23^,^29^,^30^. Although it has been argued that 4-FHis may not be a good substrate for the HisRS of *E. coli* for steric reasons^30^, the results presented above indicate that the stabilities *and* the molecular properties (charge distribution, C-H bond polarity) of the tautomers of 2-fluorohistidine are remarkably similar to canonical histidine, making its substitution into biomolecules more amenable than 4-fluorohistidine. Of course, the *pK*_*a*_ of 2-fluorohistidine is dramatically altered, providing a useful mechanistic probe for proton transfer steps in biological processes.

### Studying Tautomeric Form in Proteins

For 2-fluorohistidine, the τ-tautomer is more stable in most environments, as it is for native histidine. In the crystal structure of 2-fluorohistidine-labelled anthrax protective antigen, a mixture of τ- and π-tautomers were observed, with the identified π-tautomer (His616) buried in the hydrophobic core of domain 4. This is consistent with our findings that lower dielectric environments shift the tautomeric equilibrium of 2-fluorohistidine toward the π-tautomer. Furthermore, hydrogen bonding was shown in our results to shift the equilibrium toward the π-tautomer, which also corroborates with the hydrogen-bond stabilized His616 in π form. So it is anticipated that local hydrogen bonds and dielectric environment will dictate tautomeric form of 2-fluorohistidine in proteins, as is often the case for canonical histidine in proteins. The introduction of the heavy atom (fluorine) in 2-fluorohistidine provides a distinctive marker in X-ray crystallography, allowing for histidine tautomeric form to be distinguished with low ambiguity in solved structures.

In contrast to 2-fluorohistidine, this work indicates that equilibrium tautomerism of 4-fluorohistidine is unfavorable in all environments. In all cases, the π-tautomer is favored by ~4 kcal/mol. This preference for the π-tautomer helps explain why 4-fluorohistidine could be successfully incorporated into the RNase A protein in work by Wells and co-workers^29^. Of the two active site histidines, His12 is indicated to be 100% π-tautomer^37^, while the tautomeric form of His119 during proton transfer is unknown (see our recent letter for further discussion)^60^. Having a viable enzyme with 4-FHis substitution may indicate the π-tautomer is the active form during mechanistic steps. Overall, these results indicate that fluorohistidines may be poised for studying the role of histidine tautomerization or protonation state in biological mechanisms, as the τ-tautomer of 2-fluorohistidine is more stable, but tautomeric form can easily be identified by crystallography in labeled proteins and via ^19^F and ^13^C NMR. Meanwhile the π-tautomer is strongly favored in 4-fluorohistidine, and the effect of this tautomeric stability perturbation on protein function can be evaluated.

### ^19^F NMR Spectra Reflect Protein Environment

Fluorine chemical shifts are noted for their high sensitivity to local environment. Differences in 1° and 2° structure in proteins may give rise to conformational diversity and different inductive effects on the imidazole side chain, that, in combination with varying dielectric and hydrogen bonding environments, result in the range of ^19^F signals observed for folded and unfolded proteins. For 4-fluorohistidine, differences in 1° and 2° structure in proteins may well be the primary source of changes in fluorine chemical shifts in the folded protein environment. The π-tautomer of 4-fluorohistidine appears to be quite sensitive to hydrogen bonding and inductive effects in the imidazole ring, exhibiting changes upward of 4 ppm for each perturbation. However, dielectric environment has a relatively minor effect on 4-fluorohistidine chemical shift (up to 1 ppm). All environmental effects result in much smaller fluorine chemical shift changes for both tautomers of 2-fluorohistidine: on the scale of 0.1–0.5 ppm per environmental change. This is in line with the findings of Gerig and co-workers, who postulated that diversity in fluorine chemical shifts in denatured/folded proteins arises from a sum of changes in chemical shift due to factors such as van der Waals interactions, hydrogen bonds, electric fields, and local magnetic anisotropies^61^.

### Probing Proton Transfer in Proteins

The altered *pK*_*a*_ values of fluorohistidines can be used to determine whether histidines act as general acids and bases in enzymes^29^. The differences in *pK*_*a*_ between the two isomers, with 2-fluorohistidine being less basic than 4-fluorohistidine, can be attributed primarily to solvation effects. Calculated gas phase *pK*_*a*_ values are quite similar, but the aqueous *pK*_*a*_ of 4-fluorohistidine is higher than 2-fluorohistidine. This arises from favorable solvation of the polar charge distribution of 2-fluorohistidine (τ-tautomer) that reduces the driving force for protonation. The thermally-accessible tautomeric equilibrium of 2-fluorohistidine also lowers the *pK*_*a*_ value of this isomer somewhat (up to 0.3 *pK*_*a*_ units, when tautomers are isoenergetic). Since tautomerism is not favored by 4-fluorohistidine, the *pK*_*a*_ value for this isomer is not lowered by tautomeric equilibrium. In RNase A, the *pK*_*a*_ of 4-fluorohistidines in the active site were indicated to be over 3.5 (*cf.* ~2 in aqueous solution), showing the influence of protein environment^29^.

## Conclusions

Fluorohistidines can contribute to biophysical studies of proteins in numerous ways. They have the potential to be powerful probes of tautomeric state and the contributions of tautomeric form to protein stability and enzyme mechanism. In crystallography, the fluorine atoms’ high electron density in the imidazole ring allows for definitive assignment of carbons and nitrogens, facilitating assignment of tautomeric form as well. The tautomeric stability of 2-fluorohistidine is predicted to shift with environment, with low dielectric environment shifting the tautomeric equilibrium toward the π-tautomer. While tautomeric form and tautomerism are acknowledged to be important factors in histidine’s chemical versatility and role in protein structure-function, identification of tautomeric form (at equilibrium and in rate-determining steps) has proven difficult in large biomolecular systems. 4-fluorohistidine is poised to serve as a powerful probe of the role of tautomeric form in structure and catalytic steps. In addition, its altered *pK*_*a*_ provides a tool for determining how proton transfer affects catalysis, and at pH values that are less acidic than those required for 2-fluorohistidine labeled proteins.

Additionally, biosynthetic incorporation of either fluorohistidine isomer introduces a valuable spectroscopic label for ^19^F NMR studies. Fluorine NMR spectra provide vastly simplified information relative to traditional protein NMR spectra, with the ability to report on site-specific dynamics^62^,^63^ and ligand-binding events^64^. While the origin of changes in fluorine chemical shifts are not systematically understood, this work provides some insight on how environment influences chemical shift. The ^19^F signal of 2-fluorohistidine is shown to behave “normally”—in the way that would be predicted for ^1^H chemical shifts. Deshielding is observed with hydrogen bonds, higher dielectric, and nearby electron withdrawing groups. In contrast, the ^19^F chemical shifts of 4-fluorohistidine are “reverse”, with environmental changes that normally deshield nuclei (such as hydrogen bonding, higher dielectric) resulting in shielding of the fluorine nucleus. Fluorohistidines are poised to serve as powerful biophysical probes of protein structure-dynamics-mechanisms. This work can serve as a guide for interpretation of mechanistic experiments and spectroscopic data with fluorohistidine-labeled biomolecules.

## Methods

Optimized geometries, frequencies, and NMR chemical shifts were calculated for Gly-FHis-Gly tripeptides and the following analogues, in order to simulate effects of peptide environment on fluoroimidazole ring properties: 2-fluorohistidine **(2-FHis)**, 4-fluorohistidine **(4-FHis)**, 2-fluoro-(5-methyl)-imidazole **(2F-MeIm)**, 4-fluoro-(5-methyl)-imidazole **(4F-MeIm)**, *N-*acetyl-2FHis-O-methyl ester **(NAc-2FHis-OMe)**, *N-*acetyl-4FHis-O-methyl ester **(NAc-4FHis-OMe),** 2-fluoro-(5-trifluoromethyl)-imidazole **(2F-CF**_**3**_**Im)**, 4-fluoro-(5-trifluoromethyl)-imidazole **(4F-CF**_**3**_**Im)**. All quantum chemical calculations were performed using Gaussian 09 package^65^ with calculations set up in GaussView 5^66^. BHandHLYP^67^ with 6–311++G(d, p) basis set is considered for all calculations. Initially, all the geometries were optimized to get a stable conformation, and frequencies were calculated to verify that geometries are at local energy minima. These geometries were used for calculating absolute shielding constants, energies and electrostatic potential charge distribution (by ChelpG method)^68^ at the same level of theory. Free energies were obtained from frequency calculations.

Solvation was modeled with the Self Consistent Reaction Field (SCRF) method, using the conductor polar continuum model (CPCM)^69^ to study the effect of dielectric environment/solvation on spectra and tautomeric stabilities. To estimate chemical shifts and molecular properties of buried residues (versus solvent-exposed side chains), aqueous (ε = 78.3) calculations are compared to those in tetrahydrofuran (THF, ε = 7.6) and acetonitrile (ε = 35.7) to see effects of less polar environments.

NMR shielding constants were calculated using Gauge Independent Atomic Orbital (GIAO)^70^,^71^ method, and are reported for ^13^C and ^19^F nuclei. The NMR chemical shifts (δ) reported for the compounds are calculated by the formula δ_compound_ = σ_ref_ − σ_compound_ from the shielding value (σ) of the compound and shielding of the reference compound: C_6_F_6_ for ^19^F and tetramethylsilane (TMS) for ^13^C. Shielding tensors for the reference compound are calculated with BHandHLYP/6–311++G(d, p) method after optimizing the molecule with the same method. Experimental ^13^C NMR data for 2-fluorohistidine in water and variable-temperature ^19^F NMR data for 4-fluorohistidine was collected on a Varian Mercury VX 300 MHz NMR equipped with a Varian 300 SW/BB probe.

## Additional Information

**How to cite this article**: Kasireddy, C. *et al*. The Biophysical Probes 2-fluorohistidine and 4-fluorohistidine: Spectroscopic Signatures and Molecular Properties. *Sci. Rep.* **7**, 42651; doi: 10.1038/srep42651 (2017).

**Publisher's note:** Springer Nature remains neutral with regard to jurisdictional claims in published maps and institutional affiliations.

## Supplementary Material

Supplementary Information

## Figures and Tables

**Figure 1:**
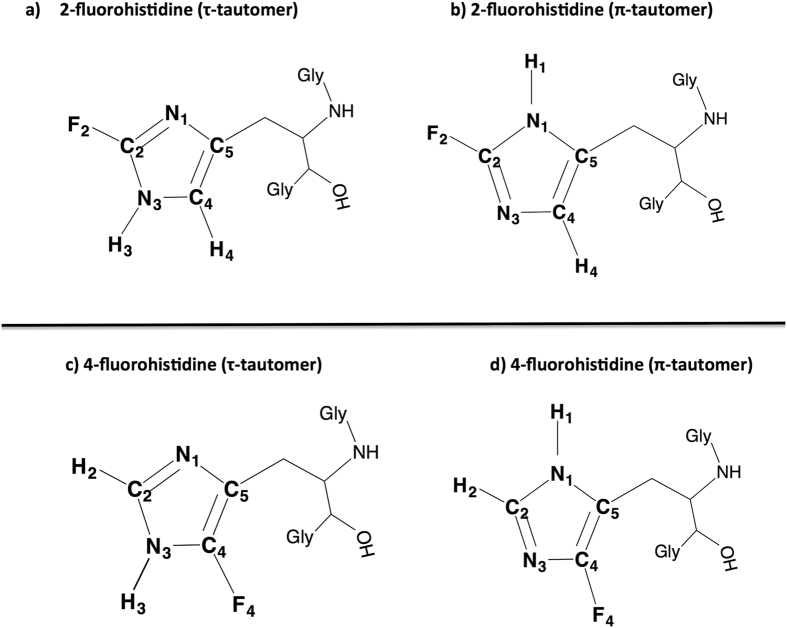
The two tautomeric forms of 2-fluorohistidine ((**a**) τ-tautomer, (**b**) π-tautomer) and 4-fluorohistidine ((**c**) τ-tautomer, (**d**) π-tautomer) in Gly-FHis-Gly tripeptide.

**Figure 2:**
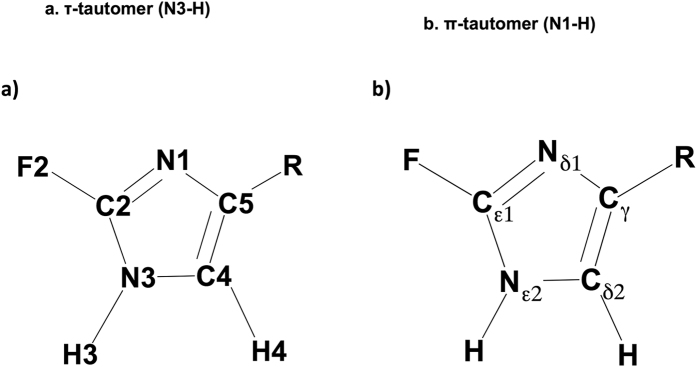
The imidazole ring of 2-fluorohistidine (τ-tautomer). The labels used for imidazole ring atoms in this work are shown in (**a**), while (**b**) provides symbols used in other literature discussions of histidine.

**Table 1:** Effect of dielectric environment on ^19^F chemical shifts (relative to C_6_F_6_) and relative tautomeric stabilities.

Molecule	Tautomer	In water (ε = 78.4)		In acetonitrile (ε = 35.7)		In tetrahydrofuran (ε = 7.4)	
		^19^F δ_ppm_	ΔE	^19^F δ_ppm_	ΔE	^19^F δ_ppm_	ΔE
**2F-tripeptide**	**τ**	53.19 ppm	0.00 kcal/mol	52.83 ppm	0.00 kcal/mol	52.91 ppm	0.00 kcal/mol
	**π**	52.22 ppm	1.70 kcal/mol	52.06 ppm	1.34 kcal/mol	51.32 ppm	1.36 kcal/mol
**4F-tripeptide**	**τ**	6.52 ppm	4.85 kcal/mol	6.48 ppm	4.74 kcal/mol	6.57 ppm	3.91 kcal/mol
	**π**	19.21 ppm	0.00 kcal/mol	19.31 ppm	0.00 kcal/mol	20.16 ppm	0.00 kcal/mol

**Table 2:** Effect of intramolecular hydrogen bonding on ^19^F chemical shifts (in ppm, relative to C_6_F_6_) and relative tautomeric energies.

Molecule	Tautomer	with intramolecular hydrogen bonding (zwitterion)		without intramolecular hydrogen bonding (capped)	
		^19^F δ_ppm_	ΔE	^19^F δ_ppm_	ΔE
**2-fluorohistidine**	**τ**	55.89 ppm	0.00 kcal/mol	55.50 ppm	0.00 kcal/mol
	**π**	52.28 ppm	1.70 kcal/mol	52.39 ppm	1.80 kcal/mol
**4-fluorohistidine**	**τ**	6.26 ppm	3.80 kcal/mol	6.83 ppm	4.69 kcal/mol
	**π**	16.89 ppm	0.00 kcal/mol	20.21 ppm	0.00 kcal/mol

Zwitterionic fluorohistidines have hydrogen bonds, while capped fluorohistidines do not.

**Table 3:** Effects of C5 substitution on tautomeric stabilities and ^19^F chemical shifts.

Molecule	-(5-methyl)-imidazole		-tripeptide		-(5-trifluoromethyl)-imidazole	
	^19^F δ_ppm_	ΔE	^19^F δ_ppm_	ΔE	^19^F δ_ppm_	ΔE
**2F-τ**	53.20 ppm	0.00 kcal/mol	53.19 ppm	0.00 kcal/mol	56.22 ppm	0.00 kcal/mol
**2F-π**	52.41 ppm	0.02 kcal/mol	52.22 ppm	1.70 kcal/mol	58.96 ppm	3.50 kcal/mol
**4F-τ**	1.71 ppm	5.62 kcal/mol	6.52 ppm	4.85 kcal/mol	21.82 ppm	2.63 kcal/mol
**4F-π**	13.62 ppm	0.00 kcal/mol	19.21 ppm	0.00 kcal/mol	36.45 ppm	0.00 kcal/mol

**Table 4:** Dipole moments of capped 2- and 4-fluorohistidine in water.

2-fluorohistidine (τ-tautomer)	2-fluorohistidine (protonated form)	4-fluorohistidine (π-tautomer)	4-fluorohistidine (protonated form)
2.89 Debye	12.04 Debye	10.09 Debye	13.51 Debye

**Table 5:** Carbon chemical shifts (in ppm, relative to TMS) for imidazole carbons in Gly-FHis-Gly tripeptide.

Gly-2FHis-Gly	(τ-tautomer)	(π-tautomer)	(protonated form)
**C5(C**_**ϒ**_)	142.8 ppm	133.5 ppm	136.0 ppm
**C4(C**_**δ2**_)	118.3 ppm	129.7 ppm	122.0 ppm
**C2(C**_**ε1**_)	157.4 ppm	156.9 ppm	153.0 ppm
**Gly-4FHis-Gly**	**(τ-tautomer)**	**(π-tautomer)**	**(protonated form)**
**C5(C**_**ϒ**_)	121.2 ppm	112.1 ppm	118.4 ppm
**C4(C**_**δ2**_)	152.8 ppm	162.3 ppm	152.4 ppm
**C2(C**_**ε1**_)	136.1 ppm	137.0 ppm	136.7 ppm
