# Supplementary material for: The Biophysical Probes 2-fluorohistidine and 4-fluorohistidine: Spectroscopic Signatures and Molecular Properties
Source: Sci Rep. 2017 Feb 15;7:42651. doi: 10.1038/srep42651 (PMC5309746; doi:10.1038/srep42651)
Supplement: Supplementary Information [file srep42651-s1.pdf]

The Biophysical Probes 2-fluorohistidine and 4-fluorohistidine: Spectroscopic Signatures and Molecular Properties

*Chandana Kasireddy, Jonathan M. Ellis, James G. Bann and Katie R. Mitchell-Koch**

Supplementary Information

Table 1. Relative stabilities of aqueous tripeptide tautomers, using different electronic structure methods. Energies are reported setting lowest-energy tautomer to zero, using free energies obtained from frequency calculations.

2-fluorotripeptide free energies in kcal/mol		
**Method**	**τ-tautomer**	**π-tautomer**
**B3LYP/6-31++G(d) (cpcm)**	0	0.09
**HF/6-311++G(d,p) (cpcm)**	0	1.51
**HF/6-31++G(d) (cpcm)**	0	1.47
**B3LYP/6-31++G(d) (smd)**	0	0.73
**BHandHLYP/6-31++G(d) (smd)**	0	0.63
**Average**	0	0.90
**4-fluorotripeptide free energies in kcal/mol**		
**Method**	**τ-tautomer**	**π-tautomer**
**B3LYP/6-31++G(d) (cpcm)**	4.32	0
**HF/6-311++G(d,p) (cpcm)**	5.06	0
**HF/6-31++G(d) (cpcm)**	4.97	0
**B3LYP/6-31++G(d) (smd)**	4.58	0
**BHandHLYP/6-31++G(d) (smd)**	5.85	0
**Average**	5.00	0

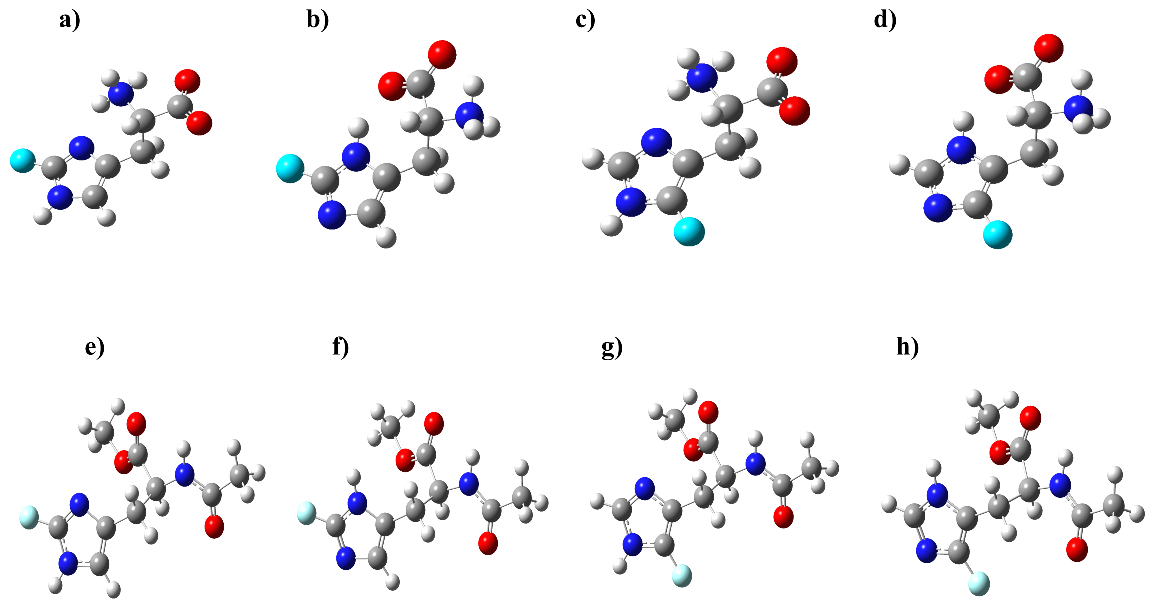

Figure S1. Lowest-energy optimized geometries for "capped" (NAc-FHis-OMe) and zwitterionic fluorohistidines: a) τ-tautomer of 2F-His b) π-tautomer of 2F-His c) a) τ-tautomer of 4F-His b) π-tautomer of 4F-His e) τ-tautomer of capped 2F-His f) π-tautomer of capped 2F-His g) τ-tautomer of capped 4F-His h) π-tautomer of capped 4F-His. Carbons are coloured grey, hydrogens white, nitrogens blue, oxygens red, and fluorines aqua.

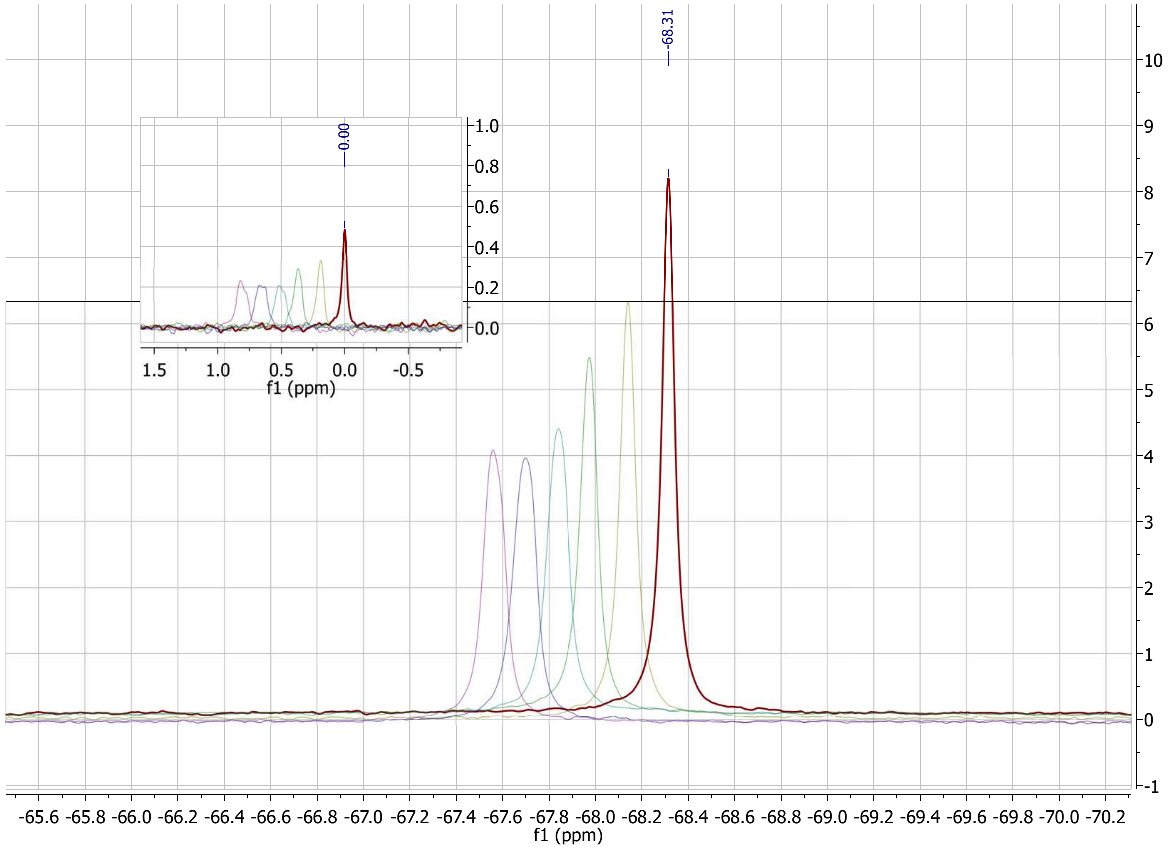

Figure S2. Variable Temperature NMR of 4-fluorohistidine in H_2O. Sample was internally referenced using a 0.01% TFA/D_2O coaxial insert, and referenced to TFA at 25°C (TFA shifts shown in insert). Samples were not reference-shifted for subsequent temperature increases, only re-shimmed. The following is a table of the chemical shifts both with respect to the 25°C TFA peak (as shown in the figure), as well as relative to the thermally shifted peaks of TFA ("temperature-dependent ref.", calculated in post analysis).

Temp (^{o}C)	TFA (ppm, 25 ^{o}C ref.)	4F-His (ppm, 25 ^{o}C ref.)	4F-His (ppm, temperature- dependent ref.)
25	0	-68.31	-68.31
35	0.19	-68.14	-68.33
45	0.37	-67.97	-68.34
55	0.52	-67.86	-68.38
65	0.67	-67.68	-68.35
75	0.82	-67.55	-68.37

Table 2. Calculated pKa differences and solvation energies (ZPE) between fluorohistidine isomers in kcal/mol.

Basicity	capped 2FHis		capped 4FHis	
**gas phase basicity** $[E_{FHis^+(g)}-E_{FHis(g)}]$	-59.99 kcal/mol		-60.17 kcal/mol	
**aqueous basicity** $[E_{FHis^+(aq)}-E_{FHis(aq)}]$	-28.79 kcal/mol		-30.23 kcal/mol	
**solvation energy** $[E_{(aq)}-E_{(g)}]$	**2FHis (τ)**	**2FHis (protonated)**	**4FHis (π)**	**4FHis (protonated)**
	-13.22 kcal/mol	-54.28 kcal/mol	-14.57 kcal/mol	-56.90 kcal/mol

Table 3. Carbon chemical shifts (in ppm, TMS reference) for imidazole carbons in aqueous capped 2-fluorohistidine and 4-fluorohistidine.

capped 2FHis	(τ-tautomer)	(π-tautomer)	(protonated form)
**C5(CΥ)**	141.4 ppm	133.1 ppm	137.0 ppm
**C4(Cδ2)**	118.9 ppm	130.2 ppm	121.7 ppm
**C2 (Cε1)**	157.1 ppm	157.2 ppm	153.5 ppm
**capped 4FHis**	**(τ-tautomer)**	**(π-tautomer)**	**(protonated form)**
**C5(CΥ)**	120.0 ppm	112.1 ppm	119.8 ppm
**C4(Cδ2)**	153.3 ppm	162.4 ppm	151.3 ppm
**C2 (Cε1)**	136.1 ppm	137.5 ppm	137.5 ppm
